# Kaempferol ameliorates gestational diabetes mellitus by regulating STAT3-associated AIM2 inflammasome activation and ferroptotic injury in trophoblasts

**DOI:** 10.3389/fphys.2026.1857741

**Published:** 2026-09-22

**Authors:** Ronghui Liu, Qiqi Liu, Lanjie Yi

**Affiliations:** 1 Department of Obstetrics, Yantaishan Hospital, Yantai, Shandong, China; 2 Pharmacy Department, Qingdao Traditional Chinese Medicine Hospital, Qingdao Hiser Hospital Affiliated of Qingdao University, Qingdao, Shandong, China; 3 Institute of Literature in Chinese Medicine, Nanjing University of Chinese Medicine, Nanjing, Jiangsu, China

**Keywords:** AIM2 inflammasome, ferroptosis, gestational diabetes mellitus, kaempferol, STAT1/IRF1, STAT3

## Abstract

**Introduction:**

To investigate the protective effects of kaempferol (KPF) against gestational diabetes mellitus (GDM) and elucidate its underlying molecular mechanisms.

**Methods:**

A GDM rat model was established to assess metabolic abnormalities, inflammation, oxidative stress, and placental injury following KPF treatment. *In vitro*, high glucose (HG)-stimulated HTR-8/SVneo trophoblast cells were used to investigate the protective mechanisms of KPF. Mitochondrial dysfunction, lipid peroxidation, ferroptosis-related alterations, and AIM2 inflammasome activation were evaluated. STAT3 knockdown and AIM2 overexpression were further performed to determine their roles in HG-induced trophoblast injury.

**Results:**

KPF significantly improved glucose and lipid metabolism, reduced inflammatory cytokine levels, alleviated placental injury, and decreased oxidative stress in GDM rats. In HG-stimulated trophoblasts, KPF attenuated mitochondrial dysfunction, lipid peroxidation, and ferroptosis-related alterations by restoring GPX4 and SLC7A11 expression and inhibiting AIM2 inflammasome activation. Mechanistically, KPF suppressed STAT3 activation, while STAT3 knockdown reduced STAT1/IRF1 activation and AIM2 expression under hyperglycemic conditions. AIM2 overexpression aggravated ferroptosis-related changes and inflammatory responses.

**Conclusion:**

KPF alleviates GDM-associated placental dysfunction by modulating STAT3-associated signaling and AIM2 inflammasome activation, thereby reducing ferroptosis-related injury and inflammatory activation in trophoblasts. These findings provide new insights into the mechanisms of GDM and support KPF as a potential therapeutic candidate.

## 1 Introduction

Gestational diabetes mellitus (GDM) is a common metabolic disorder that occurs during pregnancy, characterized by compromised glucose tolerance or elevated blood sugar levels that are initially detected during this period (Goyal et al., 2022). The manifestation of GDM not only has lasting detrimental effects on maternal health—significantly increasing the risk of type 2 diabetes and cardiovascular diseases—but also adversely affects fetal development, leading to complications such as macrosomia, preterm birth, and neonatal metabolic disorders (Muche et al., 2020). Despite significant advancements in the diagnosis and management of GDM in recent years, its precise pathogenesis remains poorly understood, substantially hindering the development of targeted therapeutic strategies. Therefore, investigating the molecular mechanisms underlying GDM and identifying potential therapeutic targets is of critical importance.

In the state of GDM, hyperglycemia may have adverse effects on trophoblast cells, such as leading to dysfunction of trophoblast cells, increased apoptosis, etc., which may affect the normal development and function of the placenta (Bari et al., 2016). Trophoblasts are essential components of the placenta, playing a direct role in placental development and fetal growth. Research findings indicate that elevated glucose concentrations characteristic of GDM disrupt mitochondrial function within trophoblasts, leading to an overproduction of reactive oxygen species (ROS) that ultimately causes cellular injury and dysfunction (He et al., 2021; Shen et al., 2021). This damage ultimately impairs placental function, thereby exacerbating the pathological progression of GDM. Trophoblasts are essential components of the placenta, and their function directly influences placental development and fetal growth. Studies have shown that the hyperglycemic environment in GDM can impair trophoblast function through various mechanisms, including mitochondrial damage, oxidative stress, and inflammatory responses (Zhou et al., 2024).

Among the various types of programmed cell death, ferroptosis is distinguished as an iron-dependent process characterized by lipid peroxidation, which ultimately leads to membrane disruption and cellular dysfunction (Tang and Kroemer, 2020). Ferroptosis is closely linked to mitochondrial dysfunction, ROS generation, and metabolic disturbances. Studies have shown that the hyperglycemic environment in GDM can induce ferroptosis-related alterations in trophoblasts, leading to further placental damage and exacerbating the pathological progression of GDM (Zhao et al., 2023). Consequently, ferroptosis is likely a significant contributor to the pathogenesis of GDM and represents a promising therapeutic target. The Absent in Melanoma 2 (AIM2) inflammasome has emerged as an important regulator of inflammatory responses and has been increasingly implicated in metabolic stress-related cellular injury, including ferroptosis-associated processes. The AIM2 inflammasome is a cytosolic protein complex that responds to cellular stress, including oxidative stress, and initiates an inflammatory response (Lugrin and Martinon, 2018). The activation of the AIM2 inflammasome has been associated with various pathological conditions, including autoimmune diseases, cancer, and metabolic disorders. Emerging evidence suggests that AIM2 inflammasome activation may contribute to trophoblast injury under metabolic stress and may interact with ferroptosis-related pathways during the progression of GDM (Wang et al., 2019). Nevertheless, the precise role of the AIM2 inflammasome in GDM remains unclear, necessitating further investigations to clarify its potential as a therapeutic target.

Kaempferol (KPF), a natural flavonoid compound known for its antioxidant, anti-inflammatory, and anti-tumor properties, has attracted attention due to its ability to modulate various signaling pathways. Recent studies have further demonstrated that KPF can regulate various signaling pathways to reduce oxidative stress, inhibit inflammatory responses, and promote cellular metabolic recovery (Periferakis et al., 2022). In the context of GDM, KPF may regulate trophoblast function, inhibit ferroptosis, and enhance placental function, thereby mitigating the pathological progression of GDM through its antioxidant and anti-inflammatory properties. Moreover, KPF may exert its beneficial effects by modulating the signal transducer and activator of transcription 3 (STAT3) pathway. STAT3, a crucial signaling molecule, is involved in inflammation, immune regulation, and cellular metabolism (Zhou et al., 2023). Activation of the STAT3 pathway is closely associated with the development of numerous diseases. Given that AIM2 activation is closely associated with inflammatory transcriptional regulation, including STAT1/IRF1 signaling, STAT3 may be involved in the regulation of AIM2-associated inflammatory responses through interconnected signaling networks rather than direct transcriptional control. This potential regulatory relationship provides new insights into the molecular mechanisms underlying the protective effects of KPF in GDM. This research aims to evaluate the therapeutic efficacy of KPF in managing GDM using both *in vivo* and *in vitro* experimental models. It investigates whether KPF protects trophoblasts from hyperglycemia-induced injury by regulating STAT3-associated AIM2 inflammasome activation, ferroptosis-related alterations, and inflammatory responses.

## 2 Materials and methods

### 2.1 Animals

A total of 45 ten-week-old Sprague-Dawley rats were used in this study, including 30 females and 15 males. All rats were housed in a specific pathogen-free (SPF) laboratory environment. Male and female rats were housed separately in cages, with a density of five rats per cage. The rats were randomly assigned to five groups (n=5 per group): the Control group, the GDM group, the low-dose KPF (L-KPF) group, the medium-dose KPF (M-KPF) group, and the high-dose KPF (H-KPF) group. Prior to mating, the control group was fed a standard diet, while the other four groups were given a high-fat, high-sugar diet for six consecutive weeks. After the dietary intervention, female and male rats were housed together at a ratio of 2:1 for mating. The presence of a vaginal plug was considered indicative of gestational day 1 (GD1). Upon confirmation of pregnancy, rats were fasted for 8 hours and then intraperitoneally injected with streptozotocin (STZ) at a dose of 25 mg/kg to induce the GDM model. On gestational day 4 (GD4), fasting blood glucose (FBG) levels were measured, with an FBG>6.7 mmol/L considered indicative of successful GDM induction (Chen et al., 2022). The animal experiment was approved by the Animal Ethics Committee (Approval Number: 2023076) and conducted in strict accordance with EU regulations governing the use and care of experimental animals.

KPF (catalog no. K107145, Aladdin Biochemical Technology Co., Ltd.) was initially dissolved in dimethyl sulfoxide (DMSO, Corning) and subsequently diluted with normal saline to a final concentration of 5 mg/mL, ensuring that the DMSO concentration remained below 1% (v/v). Rats in the L-KPF, M-KPF, and H-KPF groups received KPF by gavage at doses of 50, 100, and 200 mg/kg, respectively. Rats in both the control and GDM groups received equivalent volumes of normal saline. The intervention lasted for seven consecutive days. On gestational day 17 (GD17), body weights were recorded, and tail vein blood samples were collected to measure fasting blood glucose using a glucometer (ACCU-CHEK). Thereafter, the rats were euthanized by intraperitoneal injection of 1% pentobarbital sodium at a dose of 35 mg/kg. Blood, urine, and placental tissue samples were collected. Portions of the tissues were fixed in a 4% paraformaldehyde solution, while the remaining samples were snap-frozen in liquid nitrogen for subsequent analysis.

### 2.2 Enzyme-linked immunosorbent assay

Venous blood was collected from rats and allowed to stand for 30 minutes. The supernatant was then obtained by centrifugation. The levels of cholesterol (60723ES, YEASEN), high-density lipoprotein cholesterol (HDL-C, 60737ES, YEASEN), triglycerides (BC0620, Solarbio), alanine aminotransferase (ALT, JL20911, JONLNBIO), aspartate aminotransferase (AST, JL21297-48T, JONLNBIO), creatinine (ml059158, MLBIO), urea (ml095000, MLBIO), interleukin-1 beta (IL-1β, SEKR-0002, Solarbio), interleukin-18 (IL-18, SEKR-0054, Solarbio), and tumor necrosis factor-alpha (TNF-α, SEKR-0009, Solarbio) were measured using the appropriate assay kits.

The cultured cells were centrifuged to collect the supernatant, and the concentrations of IL-1β (SEKH-0002, Solarbio), IL-18 (SEKH-0028, Solarbio), TNF-α (SEKH-0047, Solarbio), and malondialdehyde (MDA, BC0025, Solarbio) were measured according to the respective kit protocols.

### 2.3 Hematoxylin-eosin staining

Tissue samples were embedded in paraffin and sectioned into continuous slices, each 3 μm thick. After sequential deparaffinization and rehydration, the sections were stained with hematoxylin-eosin solution (G1120, Solarbio) for 4 minutes. Following staining, the sections were rinsed under running tap water for 10 minutes to remove excess dye and minimize background staining. They were then differentiated in a hydrochloric acid solution for 5 seconds and washed in distilled water for 5 minutes to remove any residual acid. After dehydration through a graded series of ethanol, the sections were cleared in xylene for 5 minutes and then mounted with neutral balsam.

### 2.4 Periodic acid-Schiff staining

Following tissue embedding in paraffin, 3 μm sections were prepared, dewaxed with xylene, and gradually rehydrated through a series of ethanol solutions. The tissue sections were then incubated at room temperature with Periodic Acid Solution (C0142S-1, Beyotime) for 10 minutes. Following this, the sections were washed in water for 5 minutes, stained with Schiff reagent (C0142S-2, Beyotime) for 30 minutes, and then washed again with water. Subsequently, the cell nucleus was stained with hematoxylin solution, typically for 30 seconds, and then rinsed with running water to develop a blue color. After adequate drying, the sections were sealed with neutral gum and examined under a microscope (CX31, Olympus).

### 2.5 Dihydroethidium staining

Fresh, unfixed tissue was sectioned into 10-μm-thick slices. For section preparation, 200 μL of cleaning solution (HR9069, Biorebo) was applied and incubated at room temperature for 5 minutes. After removing the cleaning solution, 100 μL of DHE dye working solution (HR9069, Biorebo) was added, and the sections were incubated at 37°C for 60 minutes in a dark environment. Nuclear staining was performed using 4’,6-diamidino-2-phenylindole (DAPI, GDP1024, Servicebio), followed by a 10-minute incubation at room temperature. After removing the DAPI solution, the sections were washed twice with phosphate-buffered saline (PBS, ST447-1L, Beyotime) and then mounted with glycerin. Fluorescence images were captured using a fluorescence microscope.

### 2.6 Cell culture and treatment

The HTR-8/SVneo cell line (CBP61611, Cobioer) was routinely cultured in Dulbecco’s Modified Eagle Medium (DMEM, 11965118, Thermo Fisher) supplemented with 10% fetal bovine serum (FBS, A5256701, Thermo Fisher), 100 U/mL penicillin, and 100 µg/mL streptomycin (B540734, Sangong Biotech). The cultures were incubated at 37°C in a humidified environment containing 5% CO_2_. To simulate a high-glucose (HG) condition, D-glucose (G8270, Merck) was added to the medium to reach a final concentration of 25 mM. For comparison, cells in the control group were cultured in normal-glucose (NG) medium containing 5 mM glucose (Peng et al., 2018).

Part 1: To evaluate the dose-dependent effects of KPF on trophoblast cells under high-glucose conditions, HTR-8/SVneo cells were divided into five groups: the NG group, the HG group, the L-KPF group, the M-KPF group, and the H-KPF group. The NG group cells were not subjected to any manipulation. The HG, L-KPF, M-KPF, and H-KPF groups were treated with 0, 2.5, 5, and 10 μM KPF, respectively, for 48 hours (Li-fei et al., 2014).

Part 2: To investigate the regulatory relationship between KPF and AIM2 under high-glucose conditions, HTR-8/SVneo cells were divided into five groups: the NG group, the HG group, the KPF group, the AIM2-overexpression (AIM2-OE) group, and the KPF+AIM2-OE group. The NG group cells were not subjected to any manipulation. The AIM2-OE group received lentiviral transfection of AIM2-OE. The KPF group and the KPF+AIM2-OE group were treated with 10 μM KPF for 48 hours.

Part 3: To investigate whether STAT3 overexpression affects the regulatory effect of KPF on trophoblast cells under high-glucose conditions, the HTR-8/SVneo cells were divided into five groups: the NG group, the HG group, the KPF group, the STAT3-OE group, and the KPF+STAT3-OE group. The NG group cells were not subjected to any manipulation. The KPF group and the KPF+STAT3-OE group were treated with 10 μM KPF for 48 hours. The STAT3-OE group received lentiviral transfection of STAT3-OE.

Part 4: To investigate whether inhibition of the STAT3 pathway affects the role of STAT3 overexpression in trophoblast cells, HTR-8/SVneo cells were divided into three groups: the Stattic group, the STAT3-overexpression (STAT3-OE) group, and the STAT3-OE+Stattic group. All groups were treated with high glucose and 10 μM KPF for 48 hours. The STST3-OE group underwent lentiviral transfection with the STAT3 overexpression plasmid. The Stattic and STAT3-OE+Stattic groups were treated with the STAT3 inhibitor Stattic (10 μM) for 24 hours after transfection.

Part 5: To further investigate whether STAT3 regulates AIM2 activation through the STAT1/IRF1 signaling axis under hyperglycemic conditions, HTR-8/SVneo cells were divided into four groups: NG group, HG group, HG+si-NC group, and HG+si-STAT3 group. Cells in the HG, HG+si-NC, and HG+si-STAT3 groups were exposed to high glucose conditions, while the latter two groups were transfected with negative control siRNA or STAT3-specific siRNA, respectively.

### 2.7 Cell infection

Cells were infected with lentivirus at a concentration of 5×10s TU/mL (provided by Genomeditech) in the presence of 5 μg/mL polybrene, using a multiplicity of infection (MOI) of 10. Subsequently, full-length AIM2 and STAT3 sequences were inserted into the pcDNA3.1/V5-His vector to generate overexpression constructs (designated AIM2-OE and STAT3-OE), while empty vectors served as negative controls (AIM2-NC and STAT3-NC). After 24 hours of incubation, the viral medium was replaced with fresh medium, and the cells were allowed to proliferate for an additional 48 hours.

AIM2-OE: NC_000067.7 (173177105.173293606)
STAT3-OE: NC_000077.7 (100777632.100830447)

For STAT3 knockdown experiments, HTR-8/SVneo cells were transfected with STAT3-specific small interfering RNA (si-STAT3) or corresponding negative control siRNA (si-NC) using Lipofectamine 3000 transfection reagent (Thermo Fisher Scientific) according to the manufacturer’s instructions. Briefly, cells were seeded into appropriate culture plates and transfected with si-STAT3 or si-NC when reaching approximately 60–70% confluence. After 6 hours of transfection, the medium was replaced with complete culture medium, and cells were further cultured for 24 hours before subsequent experiments.

si-STAT3: 5’-CTCAGAGGATCCCGGAAATTT-3’
si-NC: 5’-CTCAGAGGATCCCGGAAATTT-3’

### 2.8 Cell counting kit-8

HTR-8/SVneo cells were cultured for 48 hours. Subsequently, 10 μL of CCK-8 reagent (C0037, Beyotime) was added to each well, followed by incubation at 37°C for 2 hours. The optical density (OD) values were measured at 450 nm using a microplate reader (SpectraMax Gemini XPS, Molecular Devices).

### 2.9 ASC specks by immunofluorescence

Cells were fixed with 4% paraformaldehyde for 15 minutes and permeabilized with 0.1% Triton X-100. After blocking with 5% bovine serum albumin (BSA) for 1 hour, the cells were incubated overnight at 4°C with an anti-ASC antibody (ab175449, 1:200, Abcam), followed by incubation with a fluorescently conjugated secondary anti-IgG antibody. Nuclei were counterstained with DAPI, and the samples were mounted using an antifade mounting medium. Images were captured using a confocal laser scanning microscope, and the percentage of ASC speck–positive cells were quantified with ImageJ software.

### 2.10 Flow cytometry

For the assessment of mitochondrial membrane potential using JC-1 staining, the JC-1 dye (T3168, Thermo Fisher) was first diluted in an appropriate buffer solution. Subsequently, 0.5 mL of the prepared working solution was added to the cell suspension. The cells were incubated for 30 minutes to facilitate dye uptake. Following the incubation period, the cells were centrifuged to remove any unbound or excess dye. After a single wash with buffer to remove residual dye, the cells were resuspended in an appropriate medium. The mitochondrial membrane potential was subsequently assessed using flow cytometry analysis.

For the determination of intracellular ROS levels, DCFH-DA (HY-D0940, MCE) was diluted 1:1000 in serum-free medium to achieve a final concentration of 10 μM. This solution was then added to the cell suspension and incubated for 30 minutes to allow for penetration and reaction with ROS. undefined After the incubation period, the cells were centrifuged to remove any unreacted dye and then washed once with buffer to ensure the complete removal of residual dye. Following resuspension in PBS, intracellular ROS levels were measured using flow cytometry.

### 2.11 Lipid peroxidation staining

Cells were gently washed once with PBS to remove any residual culture medium and treatment compounds. The cells were then incubated with 1 mL of BODIPY 581/591 C11 lipid peroxidation probe solution (S0043S, Beyotime) at 37°C for 30 minutes in the dark. After incubation, the dye solution was removed, and the cells were washed twice with PBS to eliminate any unbound probe. Fluorescence images were immediately captured using a fluorescence microscope. BODIPY 581/591 C11 exhibits red fluorescence in its reduced (non-oxidized) state and green fluorescence when oxidized. The ratio of red to green fluorescence was used to quantitatively assess the levels of lipid peroxidation.

### 2.12 Transmission electron microscope

Cells were initially fixed in 5% glutaraldehyde at room temperature for 24 hours, followed by post-fixation with 1% osmium tetroxide for 2 hours to enhance membrane electron density and contrast. The fixed samples were then dehydrated through a graded ethanol series to remove water and subsequently infiltrated with and embedded in epoxy resin. After resin polymerization, ultra-thin sections (70 nm thick) were cut using an ultramicrotome and collected on copper grids. The sections were double-stained with uranyl acetate and lead citrate. Finally, the cellular ultrastructure was examined and imaged using TEM.

### 2.13 Molecular docking analysis

The three-dimensional structure of KPF was downloaded from the PubChem database and optimized through energy minimization. The structural model of STAT3 was obtained from the Protein Data Bank (PDB) and processed by eliminating water molecules and co-crystallized ligands, followed by hydrogen addition. Docking simulations between KPF and STAT3 were carried out using AutoDock Vina, and the binding affinity was assessed according to the predicted binding energy. The most favorable binding conformation was visualized using PyMOL to characterize the interactions between KPF and STAT3 residues, including hydrogen bonding and hydrophobic contacts.

### 2.14 Real time quantitative polymerase chain reaction

Total RNA was extracted from cells using TRIzol reagent (15596026CN, Thermo Fisher), followed by reverse transcription. The mRNA expression levels of AIM2 and STAT3 were quantified using ChamQ SYBR qPCR Master Mix (Q311-02, Vazyme). The primer sequences used in this study are as follows:

AIM2: F-5’-AAGCGCTGTTTGCCAGTTAT-3’
R-5’-CACACGTGAGGCGCTATTTA-3’
STAT3: F-5’-TTTCACTTGGGTGGAGAAGG-3’
R-5’-GCTACCTGGGTCAGCTTCAG-3’
GAPDH: F-5’-GAGTCAACGGATTTGGTCGT-3’
R-5’-TTGATTTTGGAGGGATCTCG-3’

### 2.15 Western blot

Protein samples were subjected to sodium dodecyl sulfate polyacrylamide gel electrophoresis (SDS-PAGE), transferred to a polyvinylidene fluoride (PVDF) membrane, and blocked for 2 hours. Following blocking, the membranes were incubated overnight at 4°C with primary antibodies, including AIM2 (1:1000, catalog number ab93015, Abcam), Glutathione Peroxidase 4 (GPX4, 1:1000, 52455, Cell Signaling Technology, CST), solute carrier family 7 member 11 (SLC7A11, 1:1000, 23276, CST), phospho-STAT3 (p-STAT3, 1:1000, 9145, CST), STAT3 (1:1000, 12640, CST), pro-IL-1β (1:1000, 12703, CST), IL-1β (1:1000, ab283818, Abcam), cleaved caspase-1 (1:1000, 4199, CST), pro-caspase-1 (1:1000, 24232, CST), STAT1 (1:1000, 9172T, CST), phospho-STAT1 (p-STAT1, 1:1000, 9177S, CST), IRF1 (1:1000, 9172T, CST) and β-actin (1:1000, 4967S, CST). After three washes with TBST, the membranes were incubated with a secondary antibody (1:5000, ab288151, Abcam) for 2 hours. Signal detection was performed using an enhanced chemiluminescence (ECL) solution.

### 2.16 Statistical analysis

Statistical analyses were performed using SPSS software. The data are presented as the mean ± standard deviation (SD). For datasets that conform to a normal distribution and exhibit homogeneity of variance, one-way analysis of variance (ANOVA) was used to assess statistical differences, followed by Dunnett’s test for *post hoc* pairwise comparisons. In cases where the data did not meet the assumptions of normality, the Kruskal-Wallis test was used as a non-parametric alternative. A p-value of <0.05 was considered statistically significant.

## 3 Results

### 3.1 KPF improves metabolic disorders and inflammatory responses in GDM rats

During the gestation period, rats in the GDM group exhibited symptoms including unkempt fur, polyuria, lethargy, and excessive weight gain. On the 17th day of pregnancy, the rats were weighed, and it was observed that the body weight of the rats in the GDM group was significantly higher compared to that of the control group. However, following treatment with varying doses of KPF, body weight gradually decreased (**Figure 1A**). FBG measurements indicated that the GDM group exhibited elevated blood glucose levels compared to the control group, whereas KPF treatment effectively reduced blood glucose levels, demonstrating the potential glucose-regulatory effect of KPF in GDM rats (**Figure 1B**). Serum samples were collected from the rats, and lipid-related parameters were measured using ELISA. The results showed that cholesterol and triglyceride levels were elevated in the GDM group; however, these levels were significantly reduced following KPF treatment (**Figures 1C, D**). Furthermore, the HDL level was found to be lower in the GDM group but significantly increased following KPF treatment (**Figure 1E**), suggesting that KPF has the potential to improve lipid profiles in GDM rats.

**Figure 1:**
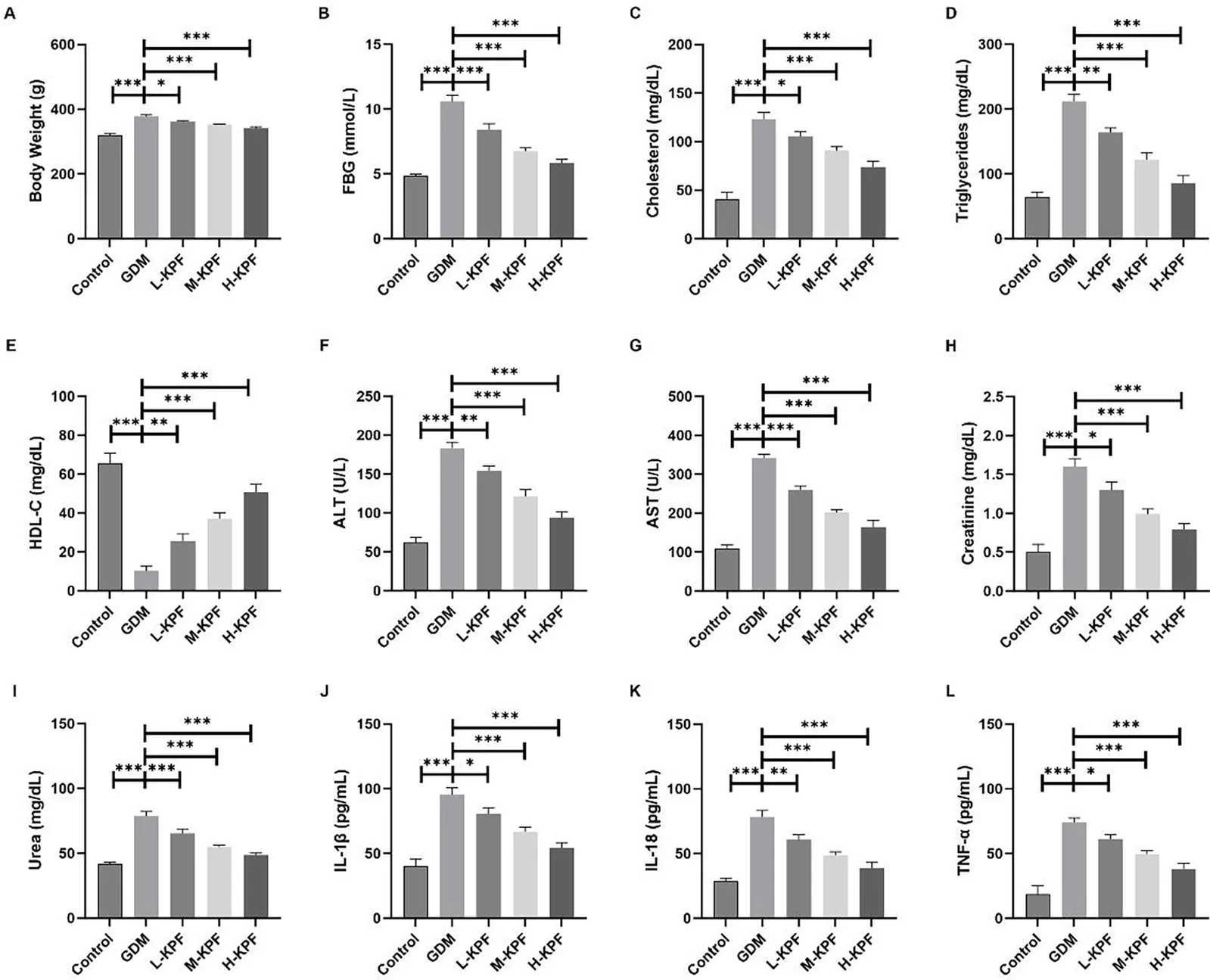
KPF ameliorates metabolic disturbances and inflammatory responses in GDM rats. **(A)** Body weight of the rats. **(B)** FBG values in rats. ELISA was performed to detect lipid indices **(C–E)**, hepatic and renal injury-related indices **(F–I)**, and inflammatory factor-related indices **(J–L)**. N = 5, ^*^P < 0.05, ^**^P < 0.01, ^***^P < 0.001.

Liver and kidney function indicators were also assessed using ELISA. The results revealed elevated levels of ALT, AST, creatinine, and urea in the GDM group. Following KPF treatment, these parameters were significantly reduced (**Figures 1F–I**), indicating that KPF attenuated GDM-associated hepatic and renal injury-related changes. Additionally, inflammatory markers were assessed, revealing that the expression levels of IL-1β, IL-18, and TNF-α were significantly elevated in the GDM group. However, these levels were significantly reduced after KPF treatment (**Figures 1J–L**), indicating that KPF effectively mitigated inflammatory responses in GDM rats. Collectively, these findings indicate that KPF ameliorates metabolic disturbances, injury-related organ changes, and inflammatory responses in GDM rats.

### 3.2 KPF ameliorates placental injury and reduces inflammation and oxidative stress in GDM rats

Histological analysis using HE staining revealed that GDM induced significant placental abnormalities in rats. These abnormalities included disorganized placental architecture, irregular arrangement of trophoblast cells, dilated and congested microvessels, and infiltration of inflammatory cells-features indicative of placental structural injury. Treatment with KPF, especially at higher doses, progressively restored placental structure, with notable improvements in chorionic villus morphology, cellular organization, and microvascular integrity, closely resembling that of the control group (**Figure 2A**). PAS staining further demonstrated disrupted glycogen metabolism in GDM rats, characterized by uneven and reduced glycogen storage in villous cells compared to controls. However, KPF treatment resulted in a dose-dependent restoration of glycogen distribution, with uniform accumulation and improved vascular morphology observed at higher doses (**Figure 2B**).

**Figure 2:**
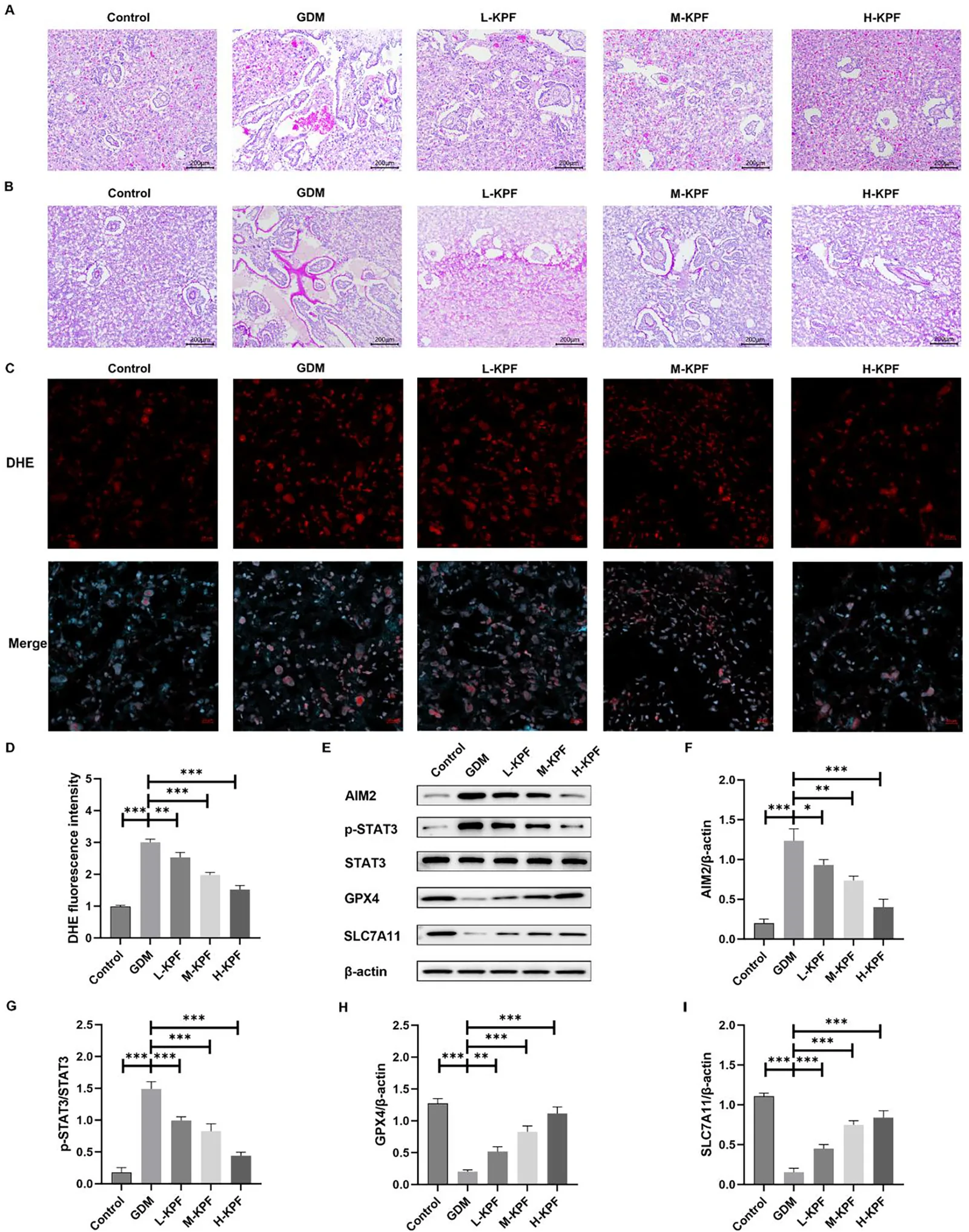
KPF ameliorates placental pathological changes and oxidative stress in GDM rats. **(A)** HE staining was performed to evaluate pathological alterations in placental tissues. Magnification: 100×; scale bar: 200 μm. **(B)** PAS staining was performed to assess glycogen distribution and deposition in placental tissues. Magnification: 100×; scale bar: 200 μm. **(C, D)** DHE staining was performed to evaluate ROS accumulation in placental tissues. Magnification: 200×; scale bar: 20 μm. **(E–I)** Western blot analysis was performed to determine the protein expression levels of AIM2, p-STAT3, GPX4, and SLC7A11 in placental tissues. N = 3, ^*^P < 0.05, ^**^P < 0.01, ^***^P < 0.001.

ROS accumulation in placental tissues was evaluated using DHE staining, revealing a significant increase in the GDM group compared to controls. Treatment with KPF resulted in a dose-dependent reduction in ROS levels, approaching normal levels at higher concentrations (**Figures 2C, D**). Western blot analysis of key proteins demonstrated that GDM upregulated the expression of AIM2 and p-STAT3 in placental tissues, suggesting enhanced AIM2-associated inflammatory signaling and STAT3 activation. Conversely, the expression levels of GPX4 and SLC7A11, key regulators involved in antioxidant defense and ferroptosis regulation, were reduced in GDM rats. KPF treatment, especially at higher doses, reversed these trends by restoring GPX4 and SLC7A11 expression and suppressing AIM2 and p-STAT3 levels. These findings suggest that KPF may alleviate GDM-induced placental dysfunction by modulating inflammatory signaling and restoring antioxidant capacity (**Figures 2E–I**). The results collectively suggest that KPF exerts protective effects against GDM-induced placental pathology by modulating inflammatory and oxidative stress responses, thereby potentially contributing to improved placental homeostasis.

### 3.3 KPF alleviates high glucose-induced inflammatory activation and ferroptosis-related alterations in trophoblast cells

The effects of KPF on the viability of HTR-8/SVneo trophoblast cells were evaluated using the CCK-8 assay. Compared with the control group, cell viability was significantly decreased in the HG group, whereas KPF treatment restored cell viability in a dose-dependent manner (**Figure 3A**). Analysis of proinflammatory cytokines in the cell supernatant revealed a marked increase in the HG group, which was significantly attenuated by KPF treatment. These findings suggest that KPF suppresses HG-induced inflammatory responses in trophoblast cells (**Figures 3B, C**).

**Figure 3:**
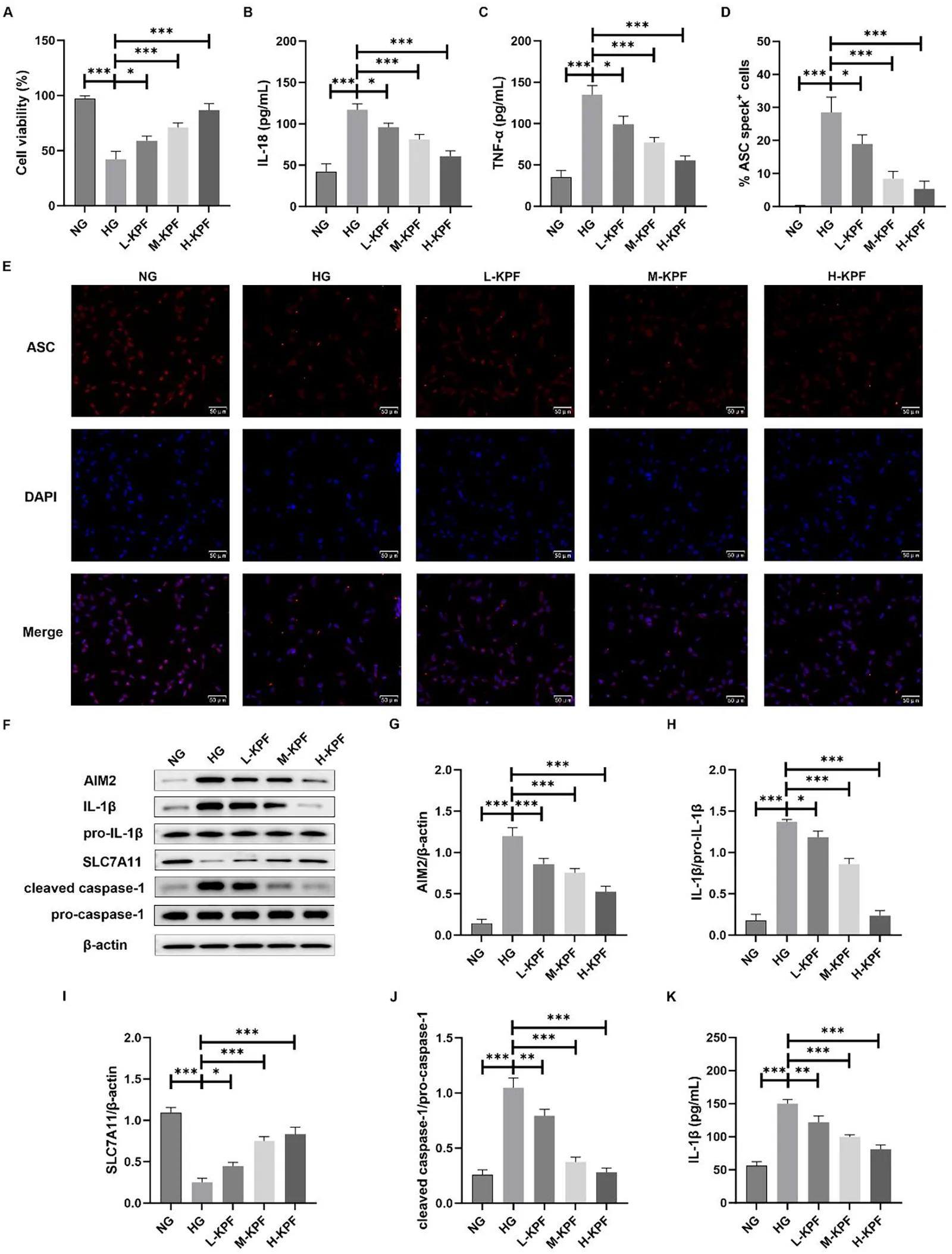
KPF alleviates high glucose-induced inflammatory activation and ferroptosis-related alterations in HTR-8/SVneo cells. **(A)** The CCK-8 assay was used to assess cell viability. **(B, C)** ELISA was used to detect the expression levels of IL-18 and TNF-α in cell supernatants. **(D, E)** Immunofluorescence staining of ASC specks. Magnification: 200×; scale bar: 50 μm. **(F-J)** Western blotting was used to detect the expression levels of AIM2, IL-1β, SLC7A11, and cleaved caspase-1. **(K)** ELISA was performed to measure IL-1β levels in cell supernatants. N = 3, ^*^P < 0.05, ^**^P < 0.01, ^***^P < 0.001.

Immunofluorescence staining of ASC revealed a significant increase in ASC speck formation in the HG group. In contrast, the proportion of ASC speck-positive cells decreased in a dose-dependent manner following KPF treatment (**Figures 3D, E**), suggesting that KPF reduces ASC speck formation and AIM2 inflammasome-associated inflammatory activation. Consistently, Western blot analysis demonstrated upregulation of AIM2, IL-1β, and cleaved caspase-1, accompanied by downregulation of SLC7A11 in the HG group, indicating activation of inflammasome-associated inflammatory signaling and changes in ferroptosis-related markers. KPF treatment restored the expression levels of these proteins in a dose-dependent manner (**Figures 3F–J**). Furthermore, ELISA results confirmed that IL-1β secretion was significantly elevated in the HG group and was effectively reduced by KPF treatment (**Figure 3K**), further supporting the anti-inflammatory effect of KPF.

Building on the observation that KPF modulates inflammasome-associated inflammatory responses, we further investigated its effects on mitochondrial function, oxidative stress, and ferroptosis in HTR-8/SVneo cells. Flow cytometry analysis of JC-1 and ROS revealed that, compared with the control group, the HG group exhibited a significant increase in JC-1 monomers, suggesting a reduction in mitochondrial membrane potential, as well as a marked elevation in intracellular ROS levels. KPF treatment promoted JC-1 aggregation, restored mitochondrial membrane potential, and significantly reduced intracellular ROS signals (**Figures 4A–D**).

**Figure 4:**
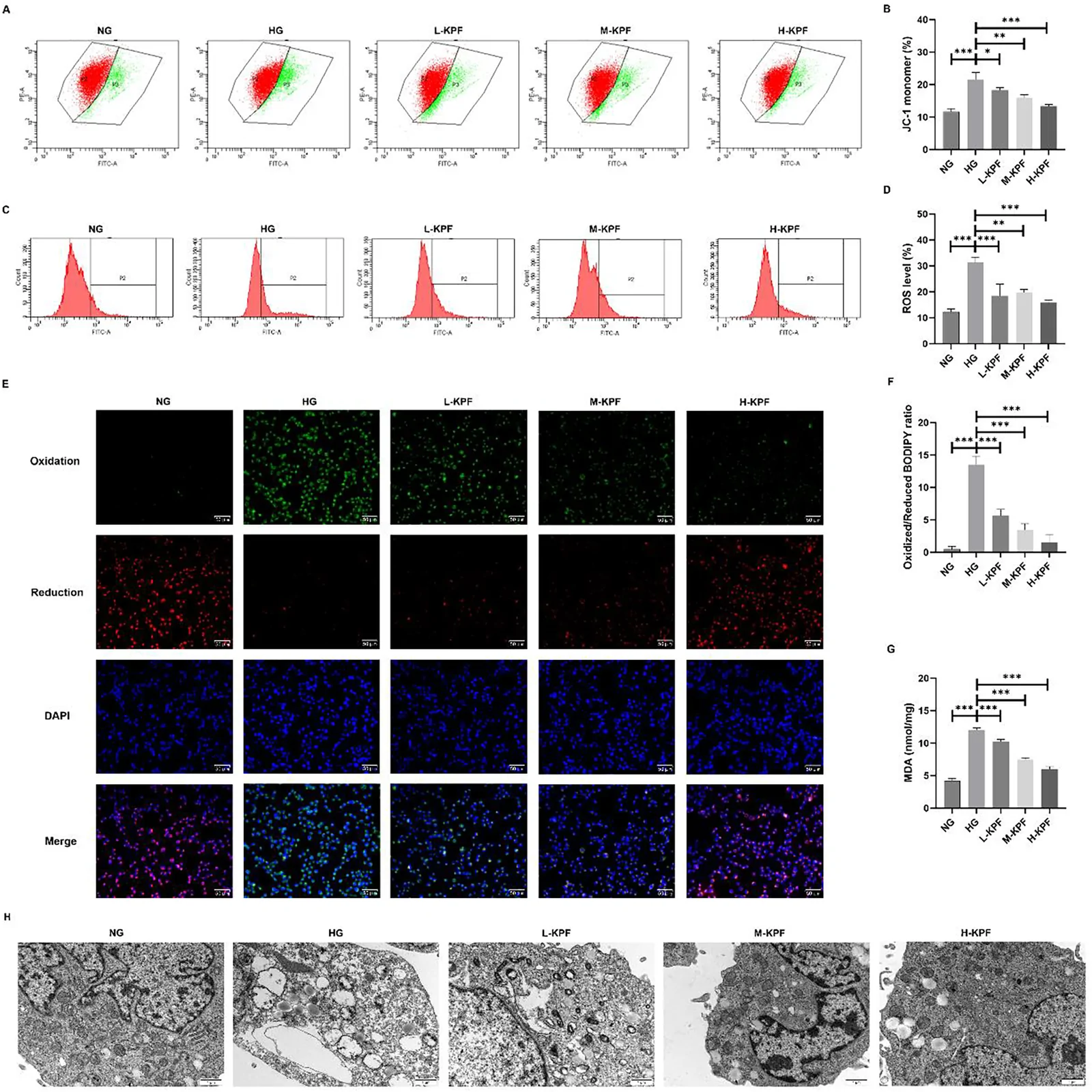
KPF alleviates mitochondrial dysfunction, oxidative stress, and ferroptosis-related alterations in trophoblast cells. Representative flow cytometry results of JC-1 staining for mitochondrial membrane potential analysis **(A)** and quantitative analysis **(B)**. Representative flow cytometry results of ROS detection **(C)** and quantitative analysis **(D)**. Representative fluorescence images of BODIPY 581/591 C11 staining for lipid peroxidation assessment **(E)** and quantitative analysis **(F)**. **(G)** Measurement of MDA levels in cells to evaluate lipid peroxidation. **(H)** TEM was used to observe mitochondrial ultrastructural changes. Magnification: 20,000×; scale bar: 1 μm. N = 3, ^*^P < 0.05, ^**^P < 0.01, ^***^P < 0.001.

BODIPY 581/591 C11 staining showed that lipid peroxidation was markedly increased in the HG group, whereas KPF treatment significantly attenuated lipid peroxidation, as reflected by changes in the red/green fluorescence ratio (**Figures 4E, F**). Consistently, measurement of MDA, a major end product of lipid peroxidation, showed that HG stimulation significantly increased intracellular MDA levels, whereas KPF treatment markedly reduced MDA accumulation (**Figure 4G**). TEM further demonstrated that mitochondria in the HG group were shrunken with disrupted cristae, while KPF treatment markedly improved mitochondrial morphology and preserved relatively intact mitochondrial structure (**Figure 4H**). Collectively, these findings indicate that KPF protects trophoblast cells from HG-induced dysfunction by ameliorating oxidative stress, mitochondrial abnormalities, and ferroptosis-related alterations, while suppressing inflammatory responses and enhancing antioxidant defense.

### 3.4 KPF attenuates AIM2 overexpression-induced ferroptosis-related alterations in trophoblast cells

The AIM2 gene was overexpressed in HTR-8/SVneo cells via lentiviral transfection, and transfection efficiency was confirmed by RT-qPCR. The AIM2-OE group exhibited a significant increase in AIM2 mRNA levels compared to the AIM2-NC group, which showed no significant changes, confirming successful transfection (**Figure 5A**). Western blot analysis of ferroptosis-related proteins showed that GPX4 and SLC7A11 expression levels were significantly reduced in both the HG and AIM2-OE groups, indicating enhanced ferroptosis-associated alterations under these conditions. Conversely, KPF treatment partially restored GPX4 and SLC7A11 expression levels in AIM2-overexpressing cells (**Figures 5B–D**). These findings indicate that AIM2 overexpression promotes ferroptosis-related alterations in trophoblast cells, whereas KPF treatment can partially alleviate these changes.

**Figure 5:**
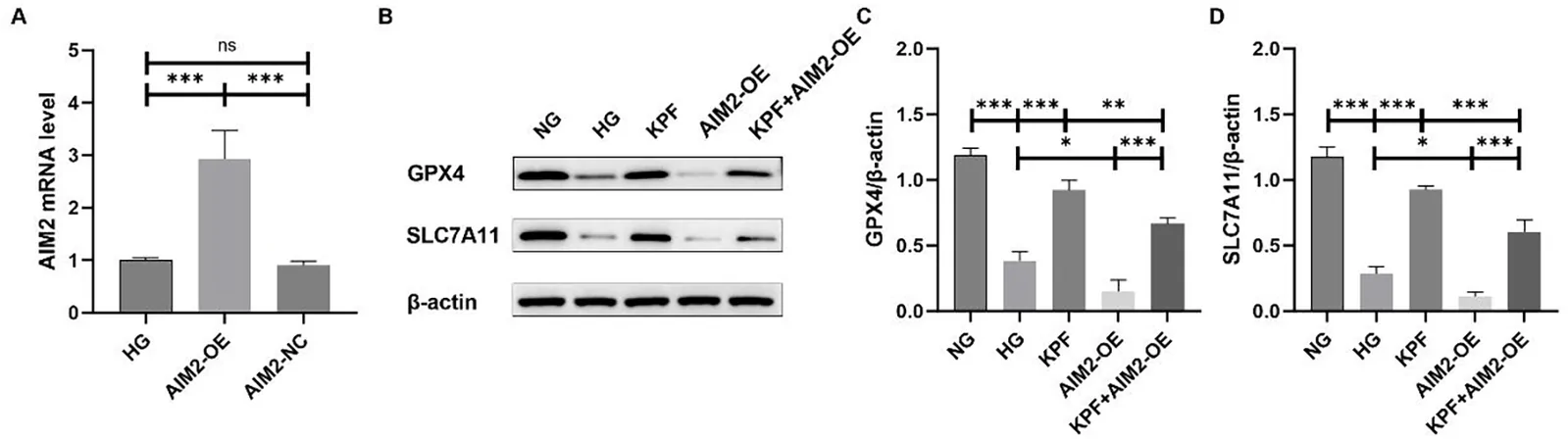
AIM2 overexpression promotes ferroptosis-related alterations in trophoblast cells. **(A)** RT-qPCR was used to assess transfection efficiency. **(B–D)** Western blot analysis of GPX4 and SLC7A11 expression across different treatment groups. N = 3, ns, not significant, ^*^P < 0.05, ^***^P < 0.001.

### 3.5 KPF protects trophoblast cells through STAT3-associated signaling regulation and modulation of inflammatory and ferroptosis-related alterations

To investigate whether STAT3 could serve as a potential molecular target of KPF, molecular docking analysis was performed. The results showed that KPF could form a stable binding mode with STAT3 protein through hydrogen bonding and hydrophobic interactions, suggesting a potential interaction between KPF and STAT3 (**Figure 6A**). The STAT3 gene was overexpressed in cells via lentiviral transfection, and transfection efficiency was confirmed by RT-qPCR, which showed a significant increase in STAT3 mRNA levels in the STAT3-OE group compared to the STAT3-NC group (**Figure 6B**). This outcome confirmed the success of the transfection. Further analysis of STAT3 mRNA across all experimental groups demonstrated that both HG and STAT3-OE conditions significantly increased STAT3 expression, which was markedly reduced following treatment with a high dose of KPF (**Figure 6C**). Additionally, the protective effect of KPF on cell viability in the STAT3-OE group was evaluated, revealing that KPF effectively counteracted the cytotoxicity induced by high glucose and STAT3 overexpression, thereby preserving cellular health (**Figure 6D**). These findings indicate that KPF is associated with the regulation of STAT3-related cellular responses under hyperglycemic stress.

**Figure 6:**
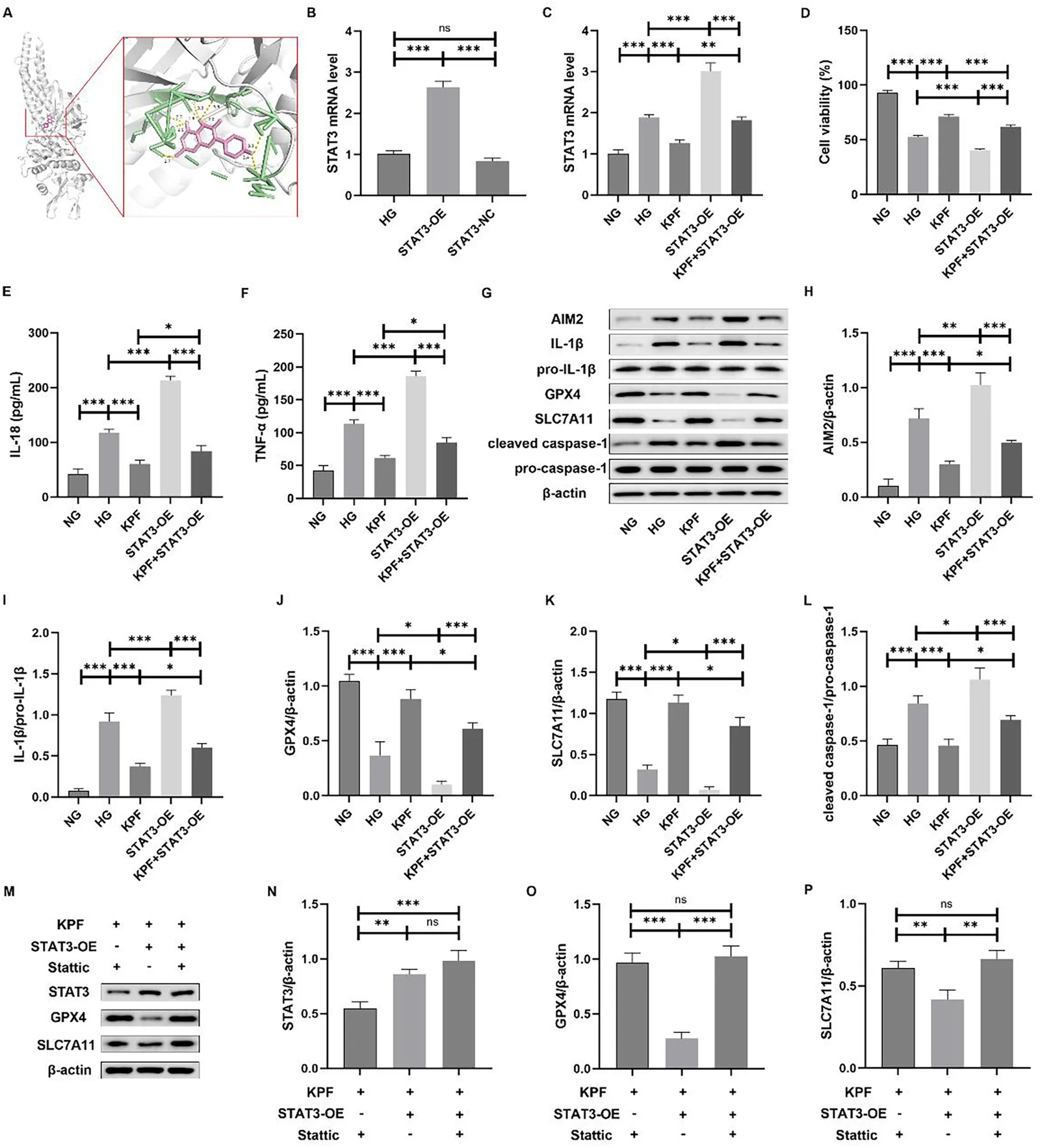
KPF modulates trophoblast inflammatory responses and ferroptosis-related alterations through STAT3-associated signaling regulation. **(A)** Molecular docking analysis showing the potential interaction between KPF and STAT3 protein. **(B, C)** RT-qPCR analysis of STAT3 mRNA expression and transfection efficiency in HTR-8/SVneo cells. **(D)** CCK-8 assay was used to evaluate cell viability. **(E, F)** ELISA quantification of inflammatory cytokines IL-18 and TNF-α in cell supernatants. **(G–L)** Western blot analysis of AIM2, IL-1β, pro-IL-1β, GPX4, SLC7A11, cleaved caspase-1, and pro-caspase-1 protein expression in cells subjected to various treatments. **(M–P)** Western blot analysis of STAT3-associated changes in STAT3, GPX4, and SLC7A11 protein expression in cells subjected to various treatments. N = 3, not significant, ^*^P < 0.05, ^**^P < 0.01, ^***^P < 0.001.

The concentrations of the inflammatory cytokines IL-18 and TNF-α in cell supernatants were quantified using ELISA. Consistent with the Western blot results, both cytokines were significantly elevated in the STAT3-OE group compared to the HG group. High-dose KPF treatment significantly reduced their expression (**Figures 6E, F**), suggesting that STAT3-associated signaling may participate in the regulation of inflammatory responses. Western blot analysis further revealed that the protein levels of AIM2, IL-1β, and cleaved caspase-1 were increased, whereas the levels of GPX4 and SLC7A11 were decreased in both the HG and STAT3-OE groups. KPF treatment reversed these changes by restoring the expression of GPX4 and SLC7A11 and by suppressing the levels of AIM2, IL-1β, and cleaved caspase-1 (**Figures 6G–L**). These findings suggest that KPF modulates STAT3-associated inflammatory activation and ferroptosis-related alterations under hyperglycemic conditions.

To investigate whether inhibition of the STAT3 pathway affects the role of AIM2 overexpression in trophoblast cells, HTR-8/SVneo cells were divided into three groups: Stattic, AIM2-OE, and AIM2-OE+Stattic. Western blot analysis revealed that AIM2 protein levels were elevated in the AIM2-OE group, accompanied by decreased expression of GPX4 and SLC7A11, as well as increased levels of IL-1β and cleaved caspase-1 levels. Stattic treatment partially reversed these changes (**Figures 6M–P**), suggesting that STAT3 inhibition may influence AIM2-associated inflammatory activation and ferroptosis-related alterations.

### 3.6 STAT3 is associated with AIM2 regulation through STAT1/IRF1-related signaling changes in trophoblast cells

To further investigate the relationship between STAT3 and AIM2 regulation under hyperglycemic conditions, HTR-8/SVneo trophoblast cells were transfected with STAT3-specific siRNA. Cells were divided into four groups: NG, HG, HG+si-NC, and HG+si-STAT3. RT-qPCR analysis showed that STAT3 expression was significantly increased in HG-treated cells compared with the NG group, whereas STAT3 knockdown markedly reduced STAT3 mRNA levels compared with the HG+si-NC group, confirming the efficiency of STAT3 silencing (**Figure 7A**). Furthermore, HG stimulation significantly elevated AIM2 mRNA expression compared with NG conditions. However, STAT3 knockdown significantly attenuated HG-induced AIM2 upregulation (**Figure 7B**), suggesting that STAT3 is associated with the regulation of AIM2 expression under hyperglycemic stress.

**Figure 7:**
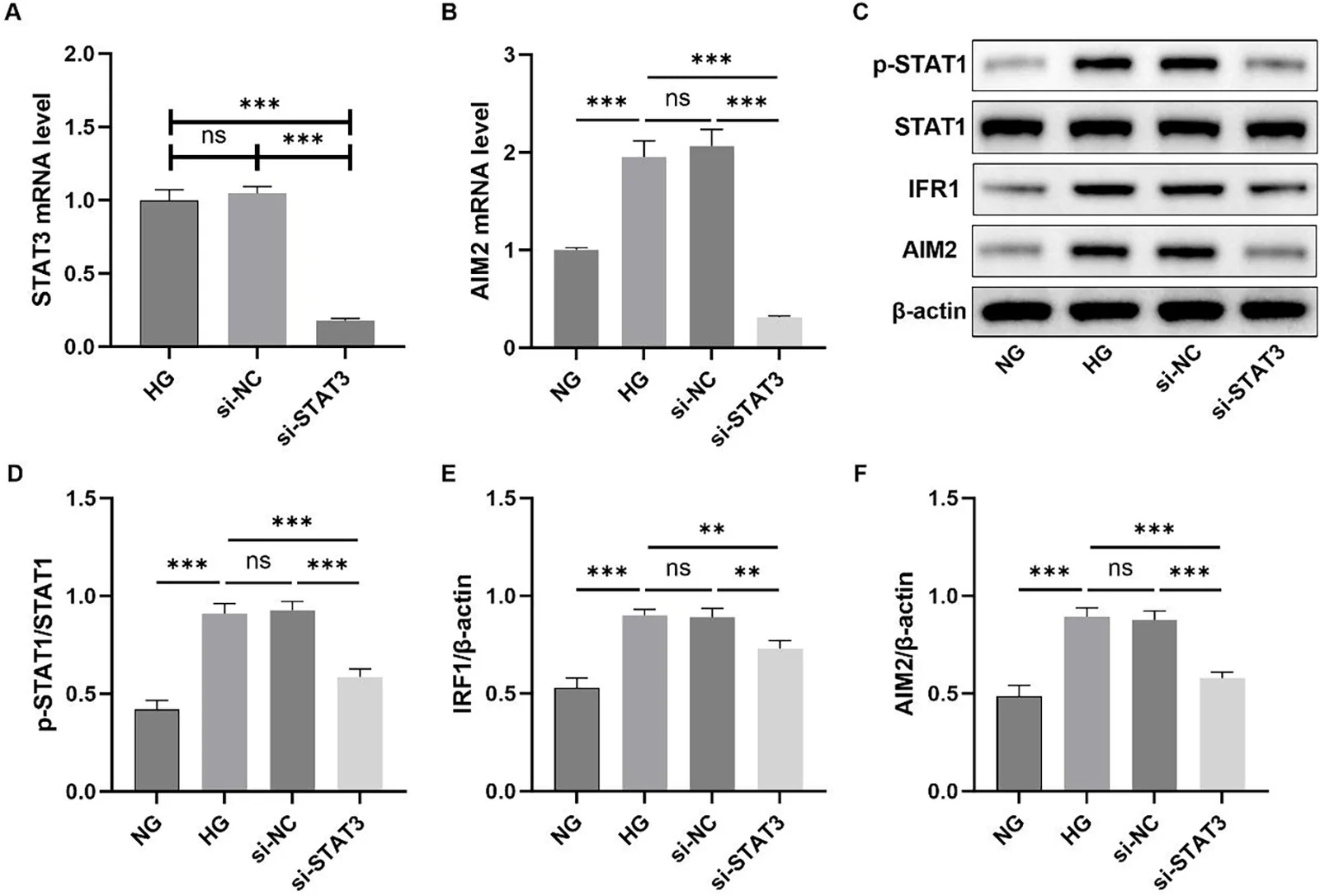
STAT3-associated changes in STAT1/IRF1 signaling and AIM2 expression in HG-stimulated trophoblast cells. **(A, B)** RT-qPCR was performed to assess STAT3 knockdown efficiency and AIM2 mRNA expression changes in HTR-8/SVneo cells subjected to different treatments. **(C–F)** Western blot analysis was performed to evaluate the expression levels of p-STAT1, STAT1, IRF1, and AIM2 proteins under different experimental conditions. N = 3, not significant, **P<0.01, ***P<0.001.

Western blot analysis revealed that HG treatment markedly increased the expression of p-STAT1, IRF1, and AIM2 compared with the NG group. These changes were further confirmed in the HG+si-NC group, while silencing STAT3 significantly reduced p-STAT1 and IRF1 levels and suppressed AIM2 protein expression compared with the HG+si-NC group (**Figures 7C–F**). These findings indicate that STAT3 knockdown is accompanied by reduced activation of STAT1/IRF1-related signaling and decreased AIM2 expression. Collectively, our data suggest that STAT3 may participate in AIM2 regulation through STAT1/IRF1-associated signaling changes in HG-stimulated trophoblast cells. However, further functional studies targeting STAT1 or IRF1 are required to establish a direct hierarchical relationship among these molecules.

## 4 Discussion

GDM is a metabolic disorder characterized by impaired glucose tolerance or hyperglycemia first diagnosed during pregnancy (Champion et al., 2022). This condition typically arises from hormonal changes during gestation that reduce insulin sensitivity, resulting in dysregulated glucose metabolism and persistent hyperglycemia (Moholdt, 2023). GDM not only impairs maternal health by increasing the risks of hypertension, polyhydramnios, and postpartum hemorrhage but also adversely affects fetal development, leading to complications such as macrosomia, preterm birth, and neonatal metabolic disorders (Martis et al., 2018). Although dietary interventions and insulin therapy are widely used, there remains an urgent need for more effective and safer targeted treatments for GDM.

In this study, KPF, a natural flavonoid, demonstrated remarkable anti-inflammatory and antioxidant activities (Imran et al., 2019) and effectively ameliorated metabolic disorders and inflammatory responses associated with GDM. *In vivo* administration of KPF significantly reduced fasting blood glucose, total cholesterol, and triglyceride levels in GDM rats, while increasing HDL cholesterol levels. KPF also improved liver function indices (ALT, AST) and kidney function indices (creatinine, urea), and markedly decreased inflammatory cytokines, indicating broad systemic metabolic protection. Notably, previous studies have reported that KPF exhibits an oral bioavailability of approximately 2% in rats, suggesting that its effective *in vivo* concentration may be limited. This finding could inform future optimization of dosing or formulation (Barve et al., 2009).

At the placental level, KPF treatment improved the histomorphology of placentas in rats with GDM. HE and PAS staining demonstrated that KPF restored villous structure, normalized glycogen deposition, and reduced microvascular congestion and inflammatory cell infiltration. DHE staining and flow cytometry analysis demonstrated that KPF reduced ROS levels in placental tissue and trophoblast cells under hyperglycemic conditions. JC-1 staining further confirmed that KPF restored the mitochondrial membrane potential, suggesting its potential to alleviate mitochondrial dysfunction in GDM. Mechanistically, high-glucose stimulation increased STAT3 activation, accompanied by enhanced STAT1/IRF1-related signaling changes and increased AIM2 expression and inflammasome activation. In contrast, KPF treatment significantly inhibited STAT3 phosphorylation and was associated with reduced STAT1/IRF1-related signaling changes and AIM2-associated inflammatory activation. STAT3 knockdown further demonstrated that STAT3 is involved in the regulation of AIM2 expression, potentially through modulation of STAT1/IRF1-related signaling rather than direct transcriptional regulation of AIM2. Furthermore, overexpression of AIM2 or STAT3 aggravated HG-induced inflammatory and ferroptosis-related alterations, whereas KPF treatment alleviated these changes, supporting the involvement of STAT3-associated signaling and AIM2-related responses in trophoblast injury under hyperglycemic conditions.

Recent studies have suggested that ferroptosis and inflammasome-mediated pyroptosis are not completely independent cell death pathways but may exhibit functional crosstalk under pathological conditions. In this study, alterations in ferroptosis-related markers, including GPX4, SLC7A11, lipid peroxidation, and mitochondrial abnormalities, were accompanied by activation of AIM2 inflammasome-related indicators, including cleaved caspase-1 and IL-1β. These findings suggest that ferroptosis and pyroptosis may interact during hyperglycemia-induced trophoblast injury. Therefore, KPF may alleviate GDM-associated placental dysfunction by simultaneously modulating oxidative damage-related responses and inflammasome-associated inflammatory activation.

Despite offering new insights into the potential role of KPF in the treatment of GDM, this study had several limitations. First, although our findings suggest that STAT3 is associated with AIM2 expression and STAT1/IRF1-related signaling changes under hyperglycemic conditions, the current data are insufficient to establish a definitive linear STAT3/STAT1/IRF1/AIM2 signaling axis. Further studies involving STAT1/IRF1 functional intervention and AIM2 loss-of-function approaches are required to clarify the hierarchical relationship and functional necessity of these molecules. Second, the lack of vehicle-matched controls, continuous food intake monitoring, and pair-fed or weight-matched animal models limits the ability to completely exclude potential effects related to solvent exposure or altered energy balance. Finally, although fluorescence-based assays were supported by complementary approaches, potential interference of flavonoid compounds with fluorescence detection cannot be completely ruled out.

## 5 Conclusions

KPF protects trophoblast cells against GDM-associated injury by modulating STAT3-associated signaling, AIM2 inflammasome activation, ferroptosis-related alterations, and inflammatory responses. These findings provide new insights into the potential mechanisms underlying GDM progression and suggest that KPF may represent a promising candidate for GDM management.

## Data Availability

The original contributions presented in the study are included in the article/**Supplementary material**. Further inquiries can be directed to the corresponding author.

## References

[B1] Bari M. F. Ngo S. Bastie C. C. Sheppard A. M. Vatish M. (2016). Gestational diabetic transcriptomic profiling of microdissected human trophoblast. J. Endocrinol. 229, 47–59. doi: 10.1530/joe-15-0424 26869332

[B2] Barve A. Chen C. Hebbar V. Desiderio J. Saw C. L. Kong A. N. (2009). Metabolism, oral bioavailability and pharmacokinetics of chemopreventive kaempferol in rats. Biopharm. Drug Dispos. 30, 356–365. doi: 10.1002/bdd.677 19722166 PMC3580176

[B3] Champion M. L. Battarbee A. N. Biggio J. R. Casey B. M. Harper L. M. (2022). Postpartum glucose intolerance following early gestational diabetes mellitus. Am. J. Obstetrics Gynecology MFM 4, 100609. doi: 10.1016/j.ajogmf.2022.100609 35272093 PMC9195159

[B4] Chen F. Ge L. Jiang X. Lai Y. Huang P. Hua J. . (2022). Construction of the experimental rat model of gestational diabetes. PloS One 17, e0273703. doi: 10.1371/journal.pone.0273703 36107823 PMC9477341

[B5] Goyal A. Gupta Y. Tandon N. (2022). Overt diabetes in pregnancy. Diabetes Therapy: Research Treat Educ. Diabetes Related Disord. 13, 589–600. doi: 10.1007/s13300-022-01210-6 35107789 PMC8991291

[B6] He H. Liu Y. Sun M. (2021). Nesfatin-1 alleviates high glucose/high lipid-induced injury of trophoblast cells during gestational diabetes mellitus. Bioengineered 12, 12789–12799. doi: 10.1080/21655979.2021.2001205 34895049 PMC8810041

[B7] Imran M. Salehi B. Sharifi-Rad J. Aslam Gondal T. Saeed F. Imran A. . (2019). Kaempferol: a key emphasis to its anticancer potential. Molecules (Basel Switzerland) 24. doi: 10.3390/molecules24122277 31248102 PMC6631472

[B8] Li-fen L. Ya-qiong C. Hai-yan H. Bo C. Nian W. (2014). Effect of kaempferol on the BaP induced apoptosis in human chorionic trophoblast cells HTR8/SV neo. Int. J. Gynecol. Obstet. 41 (6), 674–676.

[B9] Lugrin J. Martinon F. (2018). The AIM2 inflammasome: sensor of pathogens and cellular perturbations. Immunol. Rev. 281, 99–114. doi: 10.1111/imr.12618 29247998

[B10] Martis R. Crowther C. A. Shepherd E. Alsweiler J. Downie M. R. Brown J. (2018). Treatments for women with gestational diabetes mellitus: an overview of Cochrane systematic reviews. Cochrane Database Systematic Rev. 8, Cd012327. doi: 10.1002/14651858.cd012327 30103263 PMC6513179

[B11] Moholdt T. (2023). Diet, exercise and gestational diabetes mellitus. Nutrients 15. doi: 10.3390/nu15102251 37242134 PMC10223158

[B12] Muche A. A. Olayemi O. O. Gete Y. K. (2020). Gestational diabetes mellitus increased the risk of adverse neonatal outcomes: a prospective cohort study in Northwest Ethiopia. Midwifery 87, 102713. doi: 10.1016/j.midw.2020.102713 32447182

[B13] Peng H. Y. Li M. Q. Li H. P. (2018). High glucose suppresses the viability and proliferation of HTR-8/SVneo cells through regulation of the miR-137/PRKAA1/IL-6 axis. Int. J. Mol. Med. 42, 799–810. doi: 10.3892/ijmm.2018.3686 29786111 PMC6034938

[B14] Periferakis A. Periferakis K. Badarau I. A. Petran E. M. Popa D. C. Caruntu A. . (2022). Kaempferol: antimicrobial properties, sources, clinical, and traditional applications. Int. J. Mol. Sci. 23. doi: 10.3390/ijms232315054 36499380 PMC9740324

[B15] Shen D. Lu Y. Li G. Hu M. Li S. Ju H. . (2021). Mechanism of neutrophil extracellular traps generation and their role in trophoblasts apoptosis in gestational diabetes mellitus. Cell. Signalling 88, 110168. doi: 10.1016/j.cellsig.2021.110168 34634453

[B16] Tang D. Kroemer G. (2020). Ferroptosis. Curr. Biol. 30, R1292–R1297. doi: 10.1016/j.cub.2020.09.068 33142092

[B17] Wang X. Pan J. Liu H. Zhang M. Liu D. Lu L. . (2019). AIM2 gene silencing attenuates diabetic cardiomyopathy in type 2 diabetic rat model. Life Sci. 221, 249–258. doi: 10.1016/j.lfs.2019.02.035 30790610

[B18] Zhao Y. Gao Q. Li B. Wang Y. Wang Y. (2023). Ferroptosis and its potential role in gestational diabetes mellitus: updated evidence from pathogenesis to therapy. Front. Endocrinol. 14, 1177547. doi: 10.3389/fendo.2023.1177547 37664858 PMC10471987

[B19] Zhou X. Jiang Y. Wang Z. Wei L. Zhang H. Fang C. . (2024). Chemerin alleviates the placental oxidative stress and improves fetal overgrowth of gestational diabetes mellitus mice induced by high fat diet. Mol. Med. (Cambridge Mass.) 30, 239. doi: 10.1186/s10020-024-01007-2 39616329 PMC11607826

[B20] Zhou J. Liu Y. Chen J. Xiong N. Yi D. (2023). Kaempferol suppresses glioma progression and synergistically enhances the antitumor activity of gefitinib by inhibiting the EGFR/SRC/STAT3 signaling pathway. Drug Dev. Res. 84, 592–610. doi: 10.1002/ddr.22048 36852868

