# Supplementary material for: Kaempferol ameliorates gestational diabetes mellitus by regulating STAT3-associated AIM2 inflammasome activation and ferroptotic injury in trophoblasts
Source: Front Physiol. 2026 Sep 22;17:1857741. doi: 10.3389/fphys.2026.1857741 (PMC13640141; doi:10.3389/fphys.2026.1857741)
Supplement: Supplementary file 1 [file Table1.docx]

**Figure 2**

**AIM2(39 kDa)**

**GPX4(20 kDa)**

**Pro-IL-1β(31 kDa)**

**phospho-STAT3(80 kDa)**

**SLC7A11(35 kDa)**

**STAT3(80 kDa)**

**β-actin(39 kDa)**

**Figure 3**

**AIM2(42 kDa)**

**IL-1β(17 kDa)**

**pro-IL-1β(31 kDa)**

**SLC7A11(35 kDa)**

**β-actin(42 kDa)**

**Figure 4**

**GPX4(20 kDa)**

**SLC7A11(35 kDa)**

**β-actin(42 kDa)**

**Figure 5**

**AIM2(39 kDa)**

**GPX4(20 kDa)**

**IL-1β(17 kDa)**

**Pro-IL-1β(31 kDa)**

**SLC7A11(35 kDa)**

**β-actin(42 kDa)**
